# The Use of (Network) Meta-Analysis in Clinical Oncology

**DOI:** 10.3389/fonc.2019.00822

**Published:** 2019-08-27

**Authors:** Emil ter Veer, Martijn G. H. van Oijen, Hanneke W. M. van Laarhoven

**Affiliations:** Department of Medical Oncology, Cancer Centre Amsterdam, Amsterdam UMC, University of Amsterdam, Amsterdam, Netherlands

**Keywords:** meta-analysis, network meta-analysis, systematic review, oncology, gastric cancer, esophageal cancer, pancreatic cancer

## Abstract

Meta-analysis is important in oncological research to provide a more reliable answer to a clinical research question that was assessed in multiple studies but with inconsistent results. Pair-wise meta-analysis can be applied when comparing two treatments at once, whereas it is possible to compare multiple treatments at once with network meta-analysis (NMA). After careful systematic review of the literature and quality assessment of the identified studies, there are several assumptions in the use of meta-analysis. First, the added value of meta-analysis should be evaluated by examining the comparability of study populations. Second, the appropriate comparator in meta-analysis should be chosen according to the types of comparisons made in individual studies: (1) Experimental and comparator arms are different treatments (A vs. B); (2) Substitution of a conventional treatment by an experimental treatment (A+B vs. A+C); or (3) Addition of an experimental treatment (A+B vs. B). Ideally there is one common comparator treatment, but when there are multiple common comparators, the most efficacious comparator is preferable. Third, treatments can only be adequately pooled in meta-analysis or merged into one treatment node in NMA when considering likewise mechanism of action and similar setting in which treatment is indicated. Fourth, for both pair-wise meta-analysis and NMA, adequate assessment of heterogeneity should be performed and sub-analysis and sensitivity analysis can be applied to objectify a possible confounding factor. Network inconsistency, as statistical manifestation of violating the transitivity assumption, can best be evaluated by node-split modeling. NMA has advantages over pair-wise meta-analysis, such as clarification of inconsistent outcomes from multiple studies including multiple common comparators and indirect effect calculation of missing direct comparisons between important treatments. Also, NMA can provide increased statistical power and cross-validation of the observed treatment effect of weak connections with reasonable network connectivity and sufficient sample-sizes. However, inappropriate use of NMA can cause misleading results, and may emerge when there is low network connectivity, and therefore low statistical power. Furthermore, indirect evidence is still observational and should be interpreted with caution. NMA should therefore preferably be conducted and interpreted by both expert clinicians in the field and an experienced statistician. Finally, the use of meta-analysis can be extended to other areas, for example the identification of prognostic and predictive factors. Also, the integration of evidence from both meta-analysis and expert opinion can improve the construction of prognostic models in real-world databases.

## Introduction in the Use of Meta-Analysis in Oncology

In the past decade, the number of pair-wise and network meta-analyses published increased rapidly in the research field of oncology. For example, a recent study showed that there were more than 100 network meta-analyses (NMA) in oncology published between 2006 and 2015, mostly on upper gastrointestinal oncology, such as esophagogastric cancer and pancreatic cancer (1). Meta-analysis is widely recognized to be one of the highest levels of evidence in medical research (2, 3). In meta-analysis, the data of multiple studies, preferable randomized controlled trials (RCTs), that address a similar research question are pooled together. The primary aim of meta-analysis is not to create new evidence, but to establish a definitive answer to a clinical research question such as “Is treatment A more effective than B?” that was assessed in multiple studies but with inconsistent results (3, 4). Evidence even shows that meta-analysis on multiple smaller RCTs is more valuable than one large RCT (5). As there are always confounding factors in studies that can influence the outcomes, the variation of the treatment effect between the trials gives a better estimate of the mean effect than one RCT.

**Figure 1:**
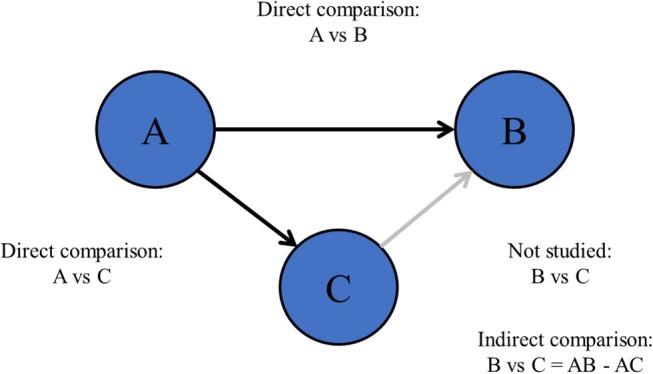
Network meta-analysis.

Also, there are more complex research questions including more than two treatments (6), such as “what is the most efficacious chemotherapy regimen for advanced esophagogastric cancer?” This type of research questions is difficult to be solved by pair-wise meta-analysis since pair-wise meta-analysis can only compare two treatments at the same time (Figure 1). Especially when comparisons between important treatments are lacking, network meta-analysis may be appropriate (7, 8).

However, the quality of a meta-analysis strongly depends on the availability of information in the individual studies, for example, a proper description of the study design and population, or the completeness of outcome data that was reported. Therefore, meta-analyses that are not adequately conducted can be very misleading and the use of meta-analysis has been criticized frequently (2, 3, 9). In this paper, we will review the most important points to decide whether the use of meta-analysis is appropriate and how to conduct meta-analysis properly in the field of oncology, with studies from upper gastrointestinal cancer as an example.

## Procedure of Conducting a Systematic Review With Meta-Analysis

### Search Strategy

Adequate systematic review is the mandatory first step of meta-analysis and can be conducted according to the PRIMA guideline (10). Preferably, a pre-specified protocol including one or more clearly defined research questions in the patient population, intervention, comparator, and outcome (PICO) structure, and clear statement of the in- and exclusion criteria should be developed and accessible to others, for example in the online PROSPERO database (11). The systematic search for studies should be conducted in multiple appropriate online databases with a search strategy that is preferably constructed by a clinical librarian, as there is evidence that the results of the search are more accurate then no clinical librarian is involved (12, 13). Additional sources, such as conference meeting abstracts can also be searched, but is hampered by the limited description on study design and possible outdated information provided in conference abstracts (14, 15).

### Data Extraction

In medical research, and especially in oncology, time-to-event data is frequently used. The hazard ratio (HR) is regarded as the most important effect-size to interpret treatment efficacy and also to include in meta-analysis. The HR is calculated using cox regression in survival analysis and indicates the relative probability of the occurrence of an event, for example death, in treatment group A vs. group B.

### Quality Assessment of Individual Studies

Within step of systematic review, also the quality of the individual studies should be evaluated before proceeding to meta-analysis (2, 9, 10). Among multiple tools to assess the quality of individual studies, the risk of bias tool developed by the Cochrane collaboration is most commonly used for RCTs (9). Two independent reviewers should rate the items with “low, high, or unknown risk of bias.” The tool can identify the most important forms of bias in studies, in example bias due to (1) systematic differences in baseline characteristics between the groups (selection bias), (2) inadequate blinding of patients and study personnel (performance bias) or (3) inadequate blinding of the person who assessed the study results (detection bias), (4) unexplained study withdrawal or incomplete outcome data (attrition bias) (16), and (5) discrepancies between reported and unreported results (reporting bias). For cohort studies in which one or more groups are compared, the Newcastle-Ottawa scale is the most frequently used tool (17). In general, it can be decided to exclude studies with poor quality beforehand, as such the pooled results of meta-analysis is less likely to be influenced by methodological quality issues of an individual study (2, 9).

### Publication Bias

**Figure 2:**
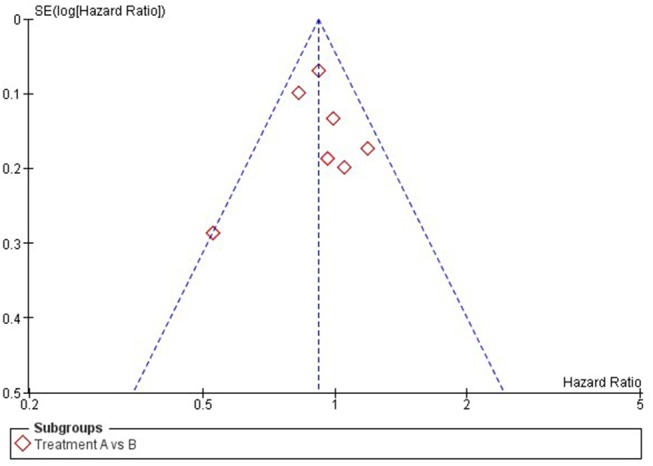
Example of a funnel plot.

Publication bias is a form of reporting bias (2, 9). For decades, it is known that studies with positive results are more likely to be published than trials with negative results (18). In addition, if trials with negative results are published, this process may have taken 2–3 times longer than publication of trials with positive results. The exact reason is still unknown. On the one hand, one could believe that journals find positive results more interesting and therefore are more willing to publish positive studies. On the other hand, there is stronger evidence that authors themselves might not publish their negative results due to a lack of time and interest to write a manuscript on negative studies (2). For both pair-wise and network meta-analysis, publication bias can be assessed using a Funnel plot, in which the effect-sizes per study are plotted against their standard error and represented as dots (see Figure 2 for an example of a Funnel plot) (19, 20). If the dots are asymmetrically scattered around the pooled effect-size (i.e., more dots on one side), then smaller studies that are tended to show larger effect-sizes may skew the pooled effect-size toward a more positive value and then some studies with more opposite results might miss in the meta-analysis (19).

## Assumptions in the Use of Meta-Analysis

### Comparability of Study Populations

When all available eligible studies with adequate quality have been selected, the added value of meta-analysis can be assessed in several steps. First the comparability of the studies should be evaluated, in order to avoid selection bias. Although the aim of the inclusion and exclusion criteria of the systematic review are meant to select comparable studies, we need to keep in mind that (almost) no study is completely identical with another (2, 5). The distribution of patient baseline characteristics can be different between the experimental and the control group, despite randomization. Furthermore, study populations can be very different even when the designs are comparable (21–23). For example, patients in RCTs on advanced esophagogastric cancer conducted in Asian countries show a longer survival time compared to patients in Western RCTs with similar design (24–27).

The pooled effect in meta-analysis may be biased when there are known prognostic or predictive unequally distributed baseline characteristics within study arms or between studies (28–31). This problem may be even larger when both RCT and cohort studies are included in meta-analysis (2). Inconsistent reporting of the baseline characteristics hampers adequate between-study comparison (32), and calls for standard sets of characteristics to report in clinical studies. This will allow more adequate comparison of patient populations between studies, such as the COMM-PACT statement for advanced pancreatic cancer trials (33).

If studies are not comparable due to substantial difference in patient populations a narrative systematic review with discussion on important clinical studies may be more valuable than a “pooled” result of a meta-analysis based on non-comparable studies (2, 4).

### Selecting the Appropriate Comparator for Meta-Analysis

Three types of comparisons can be made in comparative studies, and therefore, also in meta-analyses (see also Table 1 for clinical examples):

**Table 1:** Types of comparisons in individual studies with examples from randomized controlled trials in upper gastrointestinal cancer.

**1**	**Experimental and comparator arms are different**	
	*A* vs. *B or A+B* vs. *C*	
	Taxane-monotherapy vs. best supportive care	
	Apatinib-monotherapy vs. placebo	
	Adjuvant S-1 vs. active surveillance	
	FOLFIRINOX vs. gemcitabine-monotherapy	
	Anthracycline + cisplatin + 5-FU vs. docetaxel and cisplatin	
**2**	**Substitution of a conventional treatment by an experimental treatment**	**Substitution**
	*A+B* vs. *A+C*	B was substituted by C
	S-1 + cisplatin vs. 5-FU + cisplatin	
	Anthracycline + cisplatin + capecitabine vs. anthracycline + cisplatin + 5-FU	
**3**	**Addition of an experimental treatment**	**Added treatment**
	*A+B* vs. *B*	A was added to B
	S-1 + irinotecan vs. irinotecan-alone	S-1
	Docetaxel + cisplatin + fluoropyrimidine vs. cisplatin + fluoropyrimidine	Docetaxel

*FOLFIRINOX, 5-fluorouracil and leucovorin plus oxaliplatin and irinotecan*.

1. *Experimental and comparator arms are different treatments*. In this case the experimental and common comparator can be both single or multiple treatment entities that are all totally different or “no” treatment, such as placebo, best supportive care or active surveillance;
2. *Substitution of a conventional treatment by an experimental treatment*. The experimental regimen and common comparator regimen have the same number of agents (two or more) with similar backbone agents, but only one agent is substituted by another one;
3. *Addition of an experimental treatment*. Both the experimental and comparator regimen have similar backbones, but one agent was added to the experimental regimen.

Meta-analysis should preferably be planned according to these types of comparisons, since then the individual effect of the experimental treatment can be isolated. Previously, some pair-wise meta-analyses have been published that aimed to investigate the specific effect of one agent, for example S-1 or irinotecan as first-line treatment for advanced esophagogastric cancer and concluded that these are powerful treatment agents (34–37). However, in those meta-analyses all studies were pooled to assess irinotecan-containing vs. non-irinotecan-containing regimens (34–36) or S-1 based vs. non S-1 based regimens (37) so the specific effect of the agent of interest, could easily have been confounded by the effects of other agents in the regimens.

For meta-analysis the ideal situation is that all included studies compare their experimental treatment with one common comparator that is the golden standard in clinical practice, such as gemcitabine-monotherapy, which was the common comparator in many trials and meta-analyses on first-line treatment for advanced pancreatic cancer for more than a decade (38–40). In such cases, pair-wise meta-analysis might be sufficient to provide a definite answer to a research question. In case multiple common comparator treatments are in use for exactly the same indication, selection of studies for pair-wise meta-analysis can be more difficult. Preferably, these common comparators should be medically equal treatment choices in terms of efficacy or at least the differential effect between the common comparators should be well-known. For example, irinotecan and taxane monotherapy are both common comparators in trials on newer treatments for patients with advanced esophagogastric cancer that progressed on first-line therapy (25) and there is sufficient evidence that both treatments options are comparable in terms of efficacy (41, 42).

**Figure 3:**
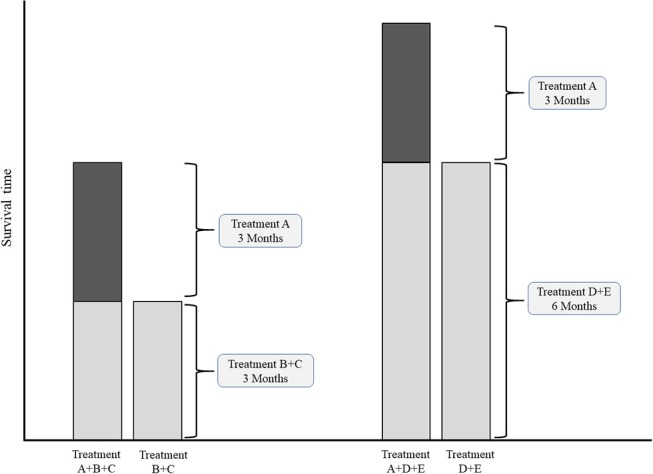
Conceptual comparisons between experimental treatment both weak and strong common comparator treatments. Left chart: comparison with relatively weak comparator. Right chart: comparisons with a relatively strong comparator.

In case of multiple common comparators, it is fair to select the strongest one for an individual study or for meta-analysis (43, 44). Imagine an addition-comparison type of study in which an experimental agent “A” was added to backbone “B” compared to the comparator regimen with the same backbone “B+C” (Figure 3). Assume that the experimental agent “A” has a fixed median overall survival of 3.0 months. If, for example the common comparator doublet regimen “B+C” (plus the natural course of the disease) is generally weak, with a median OS of 3.0 months, then the added effect of an additional agent “A” may be relatively strong (50%). On the other hand, if the doublet comparator “D+E” is generally strong, i.e., a median OS of 6.0 months, then the experimental additional agent “A” may be relatively less strong (33%). Imagine now that experimental agent “A” in combination with the less strong backbone “B+C” is used for the disease as new standard treatment. There is a chance that, if directly compared in a study, the new standard B+C might not be significantly better than the stronger comparator “D+E” which was already approved for the disease. For example, a large phase III RCT in Asian patients with advanced esophagogastric cancer showed improved OS for the addition of docetaxel to cisplatin and 5-FU (DCF) (10.5 months) compared to CF (8.5 months) (45). The authors themselves concluded that CF with median OS of 8.5 months was a weaker comparator then other regimes used in Asian populations, such as cisplatin plus S-1 with previously reported median OS of 13.0 months in the SPIRITS trial (46), or S-1 as monotherapy with median OS of 11.4, 11.0, and 10.6 months in three large RCTs (46–48). The relevance of the statistically significant effect of DCF over CF is therefore limited.

Another problem can occur when a common comparator is associated with a substantial amount of toxicity already by itself, and subsequently the addition of an experimental agent can lead to intolerability for the patient. For example in advanced esophagogastric cancer, the RILOMET-1 trial showed that rilotumumab plus epirubicin, cisplatin, and capecitabine (ECX) was significantly worse in terms of OS compared to ECX-alone, most likely due treatment intolerability which led to premature stop of treatment (49). The same applies to the V325 study, in which addition of docetaxel to the already toxic regimen CF resulted in intolerability and therefore premature treatment cessation, which negatively affected efficacy (50).

### Adequate Pooling of Treatments in Meta-Analysis

Before pooling of treatments in meta-analysis, there should be a proper overview of studies, number of direct comparisons, study and patient characteristics, and mechanism of action of all included treatments. In addition, when planning to conduct a network meta-analysis it is important to assess the geometry of the network before the actual data-analysis. Often, researchers have the tendency to merge treatment arms together in order to increase statistical power of the network or to overcome network disconnection. Plotting the network will provide more information on the relation between the treatment nodes, for example the relationship of common comparator(s) to the rest of the treatments. In general, we should consider not pool studies in pair-wise meta-analysis or merge treatments into one treatment node in NMA if:

1. the therapeutic agents have a different mechanism of action;
2. if the studies include different patient subtypes, or;
3. if the studies are conducted in a different setting.

As there three types of inadequate pooling of treatments in pair-wise meta-analysis and merging of treatments into one node in NMA happens very often, we illustrated these using some common clinical examples below.

#### Different Mechanism of Action

As for pair-wise meta-analysis, authors of various pair-wise meta-analyses on advanced esophagogastric cancer concluded that, in general, the addition of second-line targeted therapy to chemotherapy was more efficacious than chemotherapy-alone (51–54). However, since many of the targeted agents had a different mechanism of action and not all of the agents showed a positive effect in the individual studies, the results cannot be generalized to “all” second-line treatments. In this case, only the targeted agents with a similar mechanism of action should have been pooled.

In addition, many NMAs on advanced esophagogastric cancer have been published in which “targeted agents” with different mechanisms of action were merged together as one treatment node, hereby introducing bias into the network (55–57). In fact, all unique treatments should be represented as a unique node in the network, unless there is a fair reason to merge treatments that is clinically relevant and based on previous evidence. For example, in the NMA comparing all first-line chemotherapy regimens for advanced esophagogastric cancer, 5-FU, S-1, and capecitabine were merged to the class of fluoropyrimidines to increase statistical power and overcome network disconnection, but only after it was ensured that these were similar in terms of efficacy (58). This was construction of a separate subgroup NMA consisting of studies comparing 5-FU-, S-1-, and capecitabine-based regimens (with equal backbones) in a substitution-type of comparison (59). The same was done with the taxanes paclitaxel and docetaxel and the anthracyclines, epirubicin, and doxorubicin (58).

#### Different Patient Subtypes

Some of the individual studies only included a specific patient group with molecular distinct characteristics from other patients, such as human epidermal growth factor receptor 2 (HER2) or mesenchymal-epithelial transition (MET) positivity, which are treated accordingly with the HER2 (60) or MET targeted therapy (49). However, the results on the efficacy of each specific targeted therapy cannot be generalized patients with no such molecular distinct properties and vice versa. Therefore, these specific patients groups cannot be merged in pair-wise meta-analysis [such as in (52, 53)] and network meta-analyses [such as in (56, 57, 61)]. These studies cannot in fact only be pooled with or included in the same network with studies on the exact same distinct patient subgroups.

#### Different Study Setting

In other meta-analyses, studies in different treatment settings were pooled in pair-wise meta-analyses and were included and analyzed together in the same NMA, for example, studies investigating treatments for advanced esophagogastric cancer in the first-line as well as in the beyond-first line setting (51, 62). However, patients in beyond first-line studies are refractory to previous first-line treatment, and therefore pooling these two types of studies is not clinically relevant and introduced bias in the pooled estimate. The same applies for meta-analyses in which studies investigating adjuvant treatment strategies after curatively resected gastric cancer and studies investigating neoadjuvant and perioperative treatment strategies were included in the same network (55). The patients in both studies are different, as those in the adjuvant studies were only included in the trial after they had a curative resection, whereas those in de neoadjuvant or perioperative studies are not operated yet and are included in the trial straight after diagnosis. These two types of studies should therefore be compared in separate NMAs, which was done in recently published work (63).

### Adequate Assessment of Heterogeneity and Network Inconsistency

#### Definition of Heterogeneity, Transitivity, and Network Inconsistency

When an individual study with the exact same research question, i.e., the efficacy of treatment A vs. B, is repeated multiple times, the estimated effect will be different in each study by chance, which is called heterogeneity (2). In fact, none of the studies are exactly similar as there are always differences in patient- disease- or study related factors within or between studies that are confounding the estimated treatment effect (5). The amount of heterogeneity in pair-wise meta-analysis can be quantified by the *I*^2^ statistic, with a *I*^2^ percentage of 50 or more is generally seen as substantial heterogeneity (2).

**Figure 4:**
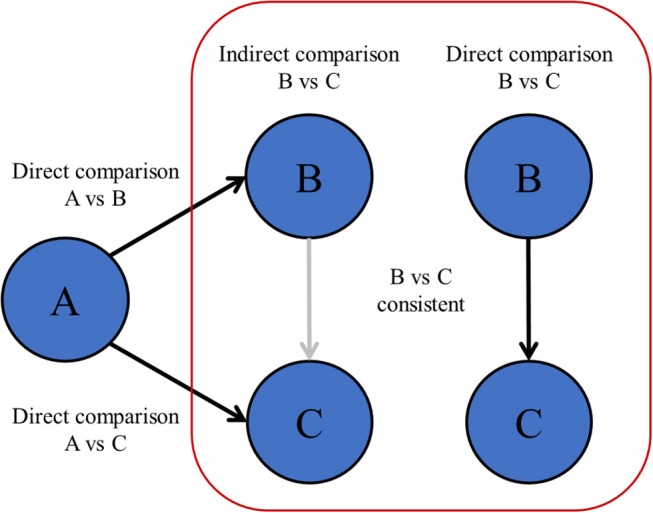
Transitivity and network inconsistency. If the direct and indirectly calculated effects are not in agreement, then there is evidence of network inconsistency, which means that the assumption of transitivity might be violated.

When comparing multiple treatments at once with NMA, indirect comparisons between treatments should be valid. This might not be the case when there are differences in patient characteristics or design between the studies as a whole that are included in the network (6, 8, 64, 65). Unlike the fact that patients are randomized between treatments within individual RCTs, patient or study characteristics of studies as a whole are not randomized between all RCTs. Therefore, RCTs can only be included in one network if all those RCTs hold similar conditions (66). The statistical manifestation of a violating the transitivity assumption is called network inconsistency, and can be observed when direct and indirect effects are incongruent within the same comparison in the network (Figure 4). Network inconsistency cannot be eliminated by randomization and is difficult to detect on sight (66). There is evidence that network inconsistency can best be identified by node-split modeling (66, 67). With this method, the complete network is split into all its closed loops of direct connections between treatments, i.e., treatment A vs. B, B vs. C, and A vs. C. Hereafter, the congruence between the directly calculated effect and the indirectly calculated effect is compared. If the direct and indirect effects are incongruent, then there may be network inconsistency in the specific closed loop (a specific set of studies). In the previous chapters, we described many sources of heterogeneity and network inconsistency, for example with inadequate pooling of treatments in pair-wise meta-analysis, and inadequate merging of treatment arms in network meta-analysis that are in fact too different.

#### Fixed- vs. Random-Effects Model

When conducting meta-analysis using a fixed-effects model, it is assumed that there is only one “true” treatment effect underlying each study, and the pooled treatment estimate is just a weighted average of the observed treatment effects of each individual study (2, 68). However, in a “random-effects” meta-analysis, it is assumed that there are multiple “true” treatment effects and that these are different among studies at random, as the reasons for different “true” effects are unknown. A pooled estimate of the treatment effect with a random-effects model therefore has a larger confidence interval than under a fixed-effect model to account for heterogeneity between “true” effects of studies, making it a more realistic estimation. In this vision, a random-effects meta-analysis is preferable as the results of meta-analysis can be generalized to a broader patient population, instead of only to the patients that were included in the individual studies (68).

#### Methods to Identify Confounders in Meta-Analysis

Sub-group analysis can be used to clarify heterogeneity in the main meta-analysis by identification of possible confounders (2, 69). First, it is wise to carefully inspect the baseline characteristics of the studies, to see whether there is an inconsistency within study arms or between studies, that can possibly influence the pooled effect. For example, in a published pair-wise meta-analysis comparing S-1 combination therapy vs. S-1 alone, sub-group analysis showed that the heterogeneity in the main analysis could be clarified by the fact that three smaller studies from China tended to overestimate the effect of S-1 combination therapy, whereas the studies conducted in Japan did not (70).

When the number of RCTs is sufficient, subgroup analysis can also be applied to NMA (6, 7, 69). For example, in a NMA comparing three important first-line chemotherapy regimens containing fluoropyrimidines capecitabine, 5-FU or S-1 for advanced esophagogastric cancer, the studies conducted in Asia and those conducted in Western countries were separately analyzed in two subgroup NMAs (59). This was done since there is evidence that the metabolism of fluoropyrimidines is different in Asian and Western patients, which might confound the overall pooled estimate in the main NMA (71). The results of the subgroup NMAs showed no difference in results and also no difference compared to the main NMA, indicating that all three fluoropyrimidines have similar efficacy in both Asian and Western patients (59).

To identify an unknown source of heterogeneity in both pair-wise meta-analysis and NMA, the influence of specific studies on the pooled outcome can be evaluated by omitting studies with deviating results or with deviating baseline characteristics in a sensitivity analysis (2, 10, 69). Otherwise, when it is unclear which study is most associated with the heterogeneity, a sensitivity analysis can be applied by omitting all included studies one by one in order to identify the studies with the largest influence on the pooled outcome and therefore may have introduced confounders in the main analysis. In case of network inconsistency that was identified using node-split models, the same approach can be used, by inspecting the baseline characteristics of the studies within the inconsistent loop and subsequently with sensitivity analyses to evaluate the extent of influence of one or more studies on the pooled outcome (67).

If no explanation for the heterogeneity or network inconsistency was found, at least it can be stated that the confounding factor is unknown despite adequate subgroup and sensitivity analysis. However, it can then also be considered not to pool the studies, but rather describe the results and discrepancies individually (2).

Of note, instead of clarification of heterogeneity, subgroup analysis can be used to identify possible predictive factors for treatment efficacy, which is important in oncology (25, 38, 69). As the effect-sizes from stratified analyses in individual studies often lack power, is can be helpful to pool this data using meta-analysis (28, 30).

#### Methods to Control for Confounding Factors

Within an individual study, a frequently used method to account for influence of unequal distributed patient characteristics on the outcome, such as survival, is to adjust the treatment effect for these characteristics using multivariable regression analysis (2, 32). However, it is not preferred to include adjusted effect-sizes in meta-analysis, as the number and type of factors for which is corrected is often different among studies, or even not reported at all (72, 73). Therefore, it is common to include the non-adjusted effect-size in meta-analysis, even though this may be biased by confounding factors.

If there are no individual patient data available; meta-regression on study level can be used to adjust for the effect of a particular confounder (73). Unfortunately, the common limitation of meta-regression is low power (72). As the effect-size of each individual study is counted as a separate data point, meta-regression might only be useful when there are at least 10 studies on a similar comparison (2).

## Strengths of Network Meta-Analysis

### Clarification of Inconsistent Outcomes From Multiple Studies Including Multiple Common Comparators

Network meta-analysis (NMA) can be helpful in case there are multiple common comparators from which the effect between all these comparators is rather unclear (6, 74). This was the case with the first-line chemotherapy for advanced esophagogastric cancer (25, 26, 75). Currently, the international guidelines make a broad recommendation for a regimen containing a platinum and a fluoropyrimidine agent with or without anthracyclines, based on evidence in over 60 RCTs (25, 27, 76, 77). However, an NMA partly clarified this inconsistency by creating an overview from best to worse available regimens. In this case, cisplatin-based cytotoxic doublets and anthracycline-platinum-fluoropyrimidine triplets showed insufficient efficacy compared to fluoropyrimidine-based doublets without cisplatin (58).

### Missing Comparisons or Weak Connections Between Important Treatments

In case of missing direct comparisons, NMA can provide insight by estimating the effect of interest indirectly (64, 65, 74). For example, two cytotoxic regimens, the CF doublet and the same regimen plus an anthracycline are standard first-line treatments for advanced esophagogastric cancer in clinical practice since 2000, but both treatments have never been compared a large RCT for almost two decades (25, 27, 77). NMA showed that there was no difference in survival between the two regimens (i.e., a hazard ratio of 1.00), which implies that the addition of an anthracycline to CF might have been redundant (58).

### Cross-Validation Between Studies and Increase of Statistical Power

In case there are a very limited number of studies, the observed pooled effect-size in pair-wise meta-analysis will be influenced by an outlier (i.e., a study that tend to over- or underestimate the treatment effect) (2). However, NMA can provide cross-validation of the observed pooled direct effect by combining direct and indirectly calculated effects from multiple other studies in the network (74, 78, 79). Also, NMA increase statistical power for weaker connections if the sample-sizes of the most important connections are sufficient (8, 64, 80). The combined effect-size becomes more precise (meaning smaller confidence intervals) than the direct pooled effect-size when the sample size increased. In sum, even when there are no missing direct comparisons between treatments of interest, NMA can still have added value over pair-wise meta-analysis. For example, in the NMA comparing the three fluoropyrimidines S-1, 5-FU, and capecitabine as first-line chemotherapy for advanced esophagogastric cancer, the three-node network was well-connected but with one weak connection between S-1 and capecitabine, which was based on only three small RCTs (59). The NMA provided cross-validation and increased power to show that all fluoropyrimidines have equal efficacy.

## Limitations of Network Meta-Analysis

Network meta-analysis (NMA) should be used in specific situations and inappropriate use of NMA can lead to misleading results.

### Limited Network Connectivity

In most research fields, such as in oncology, not all available treatments have been compared, due to time or financial restraints for example (2). Also, in the majority of cases there is only a limited number of studies comparing the exact same treatments. This can lead to limited network connectivity in NMA and therefore low statistical power (6, 74, 81). There are many examples of published NMAs with low network connectivity and large confidence intervals of the effect-sizes, making it difficult to interpret the results (82–85). For example, in a NMA on first-line palliative systemic treatments of advanced pancreatic cancer, all treatments in the network, for example gemcitabine plus nab-paclitaxel, were connected with only gemcitabine monotherapy (84).

### Limited Interpretation of Indirect Evidence

Although an indirect effect is calculated using direct comparisons in RCTs, it is still observational evidence (6, 8, 65). Indirect evidence can be helpful in case no RCT will be conducted in the future, but should be interpreted with caution (78, 86). Choosing a clinically relevant cut-off point for the effect-size may help to interpret direct but also indirect evidence. For example, there is a consensus among experts that a hazard ratio of 0.80 or less is clinically relevant for overall survival (87). Also, the ESMO clinical benefit scale states that a HR of 0.65 or lower is clinically relevant for overall survival in non-curable advanced disease (88).

### Limited Interpretation of Ranking Analysis

**Figure 5:**
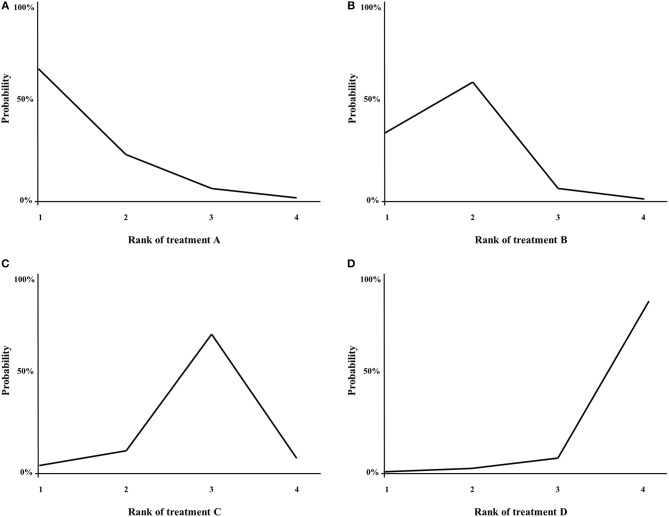
**(A–D)** Example of a SUCRA plot. X-axis: ranking of treatment. Y-axis: probability of a given treatment to be the first, second, third, or fourth best. In this example, treatment A has the largest probability to be the first best treatment.

With NMA, ranking analysis can be used to provide a better overview of which treatments are ranked on a particular outcome, such as survival prolongation, as first best, second best etc. (74, 89). In the majority of published NMAs, ranking analysis is included and graphically shown in a figure, for example a surface under the cumulative ranking area (SUCRA)-plot for one treatment outcome at once (Figure 5), or for example a heat-plot for multiple outcomes at once (90). The downside of this approach is that only the relative ranks are shown, but the absolute differences between the treatments are not (91). In example, this was the case in multiple NMAs on systemic therapy for advanced pancreatic cancer (82, 84, 85). In contrast, the relative ranking of the treatments can also be evaluated by simply categorizing the effect-sizes between all treatments and an important comparator from high to low, except that the absolute effect are still shown to the readers of the article. For example, in a NMA on first-line chemotherapy for advanced esophagogastric cancer, all treatments where categorized from highest to lowest survival benefit compared to fluoropyrimidine-monotherapy, in order to detect a certain pattern of which groups of chemotherapy classes may be better compared to others (58).

### Limited Experience of Clinicians With the Statistics of Network Meta-Analysis

Many clinicians are, to some extent, familiar with the statistical background of a pair-wise meta-analysis and can perform these analyses themselves. However, the statistical constitution of network meta-analysis is far more complex, as is the interpretation of the results derived from network meta-analysis. As nowadays more easy to use programs to conduct network meta-analysis are available, many clinicians have attempted to conduct a network meta-analysis themselves, sometimes with failure of the analysis in itself or inadequate pooling or merging of treatment arms and inadequate interpretations of results. To improve the construction of a valid network meta-analysis model and to adequately interpret its results, conducting a network meta-analysis should be done with both input from an expert clinician in the field and a statistician.

## Discussion and Future Perspectives

Meta-analysis is regarded as one of the highest sources of scientific evidence but can be misleading when it is not properly conducted (2, 4). As both the number of conventional pair-wise and NMA is rapidly increasing in the field of oncology (1, 3) we gave an overview of the most important pitfalls when conducting or interpreting meta-analysis in oncology research in the current article.

Meta-analysis can and should be used for more than aggregation of data from studies comparing treatments. In oncology, it is crucial to identify potential prognostic factors, which are patient-related characteristics with an influence on the prognosis and are not dependent of treatment (28, 30, 92). For example, patients with advanced esophagogastric cancer and metastases to the peritoneum show worse survival compared to patients with an unresectable locally advanced tumor without metastasis (93). Meta-analysis can help to select potential prognostic factors (94). Also, meta-analysis can be helpful to identify predictive factors, that indicate which patient subgroups can possibly benefit more from a certain treatment than from other treatments. Human Epidermal growth factor Receptor (HER)2-positive patients with advanced esophagogastric cancer will benefit from HER2 targeted treatment, in this case trastuzumab, but HER2-negative patients will not (25, 60). As such, HER2-status is a predictive factor for the effect of trastuzumab.

Currently, models to predict survival of an individual patient are usually based on retrospective databases or single studies, which can be both randomized studies or cohort studies. To develop a prognostic model, the factors with an independent influence on prognosis in the database or study are selected through multivariable cox regression analysis. For example, among many other predictive models for risk stratification of patients with advanced esophagogastric cancer (95–98), the Royal Marsden Hospital Index is the most common one (93). This index is based on the pooled sample size of three large RCTs and can be used to estimate the survival of patients receiving first-line palliative chemotherapy.

However, usually there are many factors that are related to the same outcome and are also somehow related to each other, which leads to heterogeneity between studies, especially the small studies (2, 99). In turn, it has been shown that here is inconsistency in the type and number of independent prognostic factors that are included in different prognostic models for a similar condition, such as advanced pancreatic (92) or esophagogastric cancer (93), usually based on small sample sizes This means that some of the non-overlapping factors may have been detected by chance. Therefore, a very large sample size is needed in order to identify all relevant factors with an independent influence on prognosis. Large real-world databases might be the only source of data with a sufficient sample size to accomplish this (100). However, as there usually is a wide variety of factors that were reported in a large real-world database, it is useful to apply a pre-selection of potential prognostic factors to focus on by conducting a systematic review and meta-analysis of the literature of previous scientific evidence from individual studies, and might also help to ensure that no potential prognostic factors are forgotten.

When possible, an independent patient data meta-analysis should be conducted (2) but this is often not possible for a variety of reasons, i.e., poor cooperation or even competition between research groups. Instead, meta-analysis on study level can also be used, in which the HRs obtained from cox regression analysis from multiple individual studies with the same prognostic factor can be pooled in order to increase statistical power (31). Preferably, the HR of the prognostic factor of interest should be controlled for exactly the same type and number of other factors in multivariable regression analysis in each individual study (72). However, often the number and type of factors that are controlled for are not reported, which was the case in multiple RCTs investigating systemic treatment for advanced pancreatic cancer (33). Therefore, the pooled result of multivariate HRs should be interpreted with caution, but might give an indication of which prognostic factors are most important to select in large real-world databases. On the other hand, the non-adjusted HRs from univariable regression analysis can safely be pooled in meta-analysis, but their effect can still be explained by other factors.

In addition, expert opinion can be used to pre-select prognostic factors through a Delphi consensus procedure, in which the experts can base their choice on both the evidence from meta-analysis and on the availability of factors in the real-world database (101–103). For example, recently a prognostic model for the survival outcome of patients with advanced esophagogastric cancer was based on data from the Dutch national cancer registry (100). The factors were pre-selected by Delphi consensus with medical oncologists according to the methodology used in a previous Delphi study in advanced pancreatic cancer (33). The evidence on certain potential prognostic factors that was shown to the experts was obtained from a previously published systematic review and meta-analysis on advanced esophagogastric cancer (31).

The same applies for obtaining more evidence for potential predictive factors for efficacy of certain treatments. The HRs of these factors, which are usually univariate, can be extracted from subgroup analysis in individual studies with a similar treatment comparison and pooled with meta-analysis (31). For example, in a published meta-analysis, the hazard ratios of stratified patient subgroups according to WHO performance status from five RCTs comparing gemcitabine-combination therapy and gemcitabine monotherapy for advanced pancreatic cancer were pooled to increase power (40). Also, another published meta-analysis pooled stratified hazard ratios of two large RCTs comparing S-1 plus cisplatin and 5-FU plus cisplatin for advanced esophagogastric cancer according to several factors (31). In turn, predictive factors may also be selected in real-world databases, if the different types of treatment are clearly defined.

In sum, the current use of meta-analysis including individual studies can be extended to other important areas, such as prognostic models, which are very important in oncology. Integration of evidence from both meta-analysis and expert opinion can improve the construction of prognostic models in real-world databases and should by applied in future studies.

## Author Contributions

All authors listed have made a substantial, direct and intellectual contribution to the work, and approved it for publication.

### Conflict of Interest Statement

The authors declare that the research was conducted in the absence of any commercial or financial relationships that could be construed as a potential conflict of interest.
